# Universal preference for Korean-type grapho-phonemic systematicity: a cross-cultural study of sound-symbol mapping in English, Chinese, and Korean speakers

**DOI:** 10.1371/journal.pone.0330674

**Published:** 2025-08-29

**Authors:** Hana Jee

**Affiliations:** Department of Language and Linguistics, York St John University, York, United Kingdom; Kyoto University of Education: Kyoto Kyoiku Daigaku, JAPAN

## Abstract

Recent studies have revealed that writing systems exhibit systematic relationships between letter shapes and their corresponding sounds, termed ‘grapho-phonemic systematicity’. This systematicity manifests differently across writing systems: Semitic languages maximize systematicity through pixel count, Chinese through perimetric complexity, and Korean through Hausdorff distance. This study investigated whether native speakers of these languages would prefer the type of systematicity found in their respective writing systems. An online survey was conducted with 845 participants (271 British, 308 Chinese, and 266 Korean) who were asked to match novel symbols from archaic writing systems with given sound pairs. Contrary to the hypothesis that participants would prefer their native writing system’s systematicity pattern, all groups showed a stronger preference for Korean-type systematicity, where similar sounds correspond to topologically similar symbols. This unexpected finding suggests that modern humans might universally prefer certain types of symbol-sound mapping, possibly influenced by institutionalized education and formal logic training. Interestingly, Korean participants showed the least preference for Korean-type systematicity, potentially due to their meta-knowledge of Hangul’s intentional design. The study reveals a disconnect between how writing systems historically evolved and what modern humans prefer, suggesting that cognitive processes in symbol-sound mapping might have been shaped by modern educational frameworks. These findings contribute to our understanding of universal cognitive principles in visual-auditory mapping and the influence of cultural and educational factors on writing system preferences.

## 1. Background

Do letter shapes have a perfectly arbitrary relationship with their sounds? A few recent studies suggest that this might not be the case. The concept of ‘grapho-phonemic systematicity’ [1] was first investigated in the context of Hangul, the Korean writing system, where letter shapes systematically represent their corresponding sounds [2]. Hangul may be the only artificial orthography of which manual and purpose of creation are explicitly documented (*Hunminjeongeum Haeryebon*, 1446). It is well known that Hangul consonants reflect the shape of the human mouth as viewed from the left side [3]—for example, ¬/g/ represents the shape of the tongue touching the soft palate. What has particularly impressed linguists, however, is the consistent and systematic visual relationship among the letters: an additional stroke indicates aspiration, and duplication signifies tense sounds. Consequently, phonemes that share the same articulation point also share visually consistent letter shapes: ¬/g/, ㅋ/k/, and ㄲ/k͈/.

By measuring the pairwise distance between phonemes and their corresponding letter shapes, we demonstrated a Pearson’s correlation coefficient of 0.6 between Hangul letters and sounds [2]. This series of studies revealed the universality of grapho-phonemic systematicity, showing significant correlations between letters and sounds across various writing systems—not only phonograms such as Arabic, Cyrillic, English, Greek, and Hebrew but also ideograms like Chinese, as well as artificial orthographies designed to substitute English alphabets, such as Pitman’s shorthand and the Shavian alphabet [1,4–8]. Notably, we found no such systematicity in fictitious writing systems created for entertainment purposes (e.g., Aurebesh or Klingon), suggesting that grapho-phonemic systematicity may result from human cultural evolution.

Different writing systems exhibited maximized grapho-phonemic systematicity depending on the metric used to measure the distances between letter shapes [1,5,8]. For example, pixel count—a simple measure of the number of pixels—yielded the most robust systematicity in Semitic writing systems such as Arabic, Hebrew, and English, indicating that more elaborate phonemes tend to be associated with larger letter shapes [1]. Certain artificial orthographies, including Hangul, demonstrated the best grapho-phonemic systematicity using the Hausdorff distance [9], a computationally sophisticated method that quantifies differences between two images [1]. This positive correlation indicates that similar-looking letters represent similar sounds. Meanwhile, the ‘characto-syllabic systematicity’ in Chinese was best captured using perimetric complexity, defined as perimeter squared divided by the sum of the ink area [10]. A recent study analysing the most frequent 1,000 Chinese characters [8] found that more complex characters tend to correspond to more elaborate syllables.

The discovery of grapho-phonemic systematicity attests to a universal cognitive preference for mapping auditory symbols to visual symbols. Although the effect size is small, the results suggest that letter shapes in conventional orthographies are not entirely arbitrary with respect to their sounds. The presence of systematicity across Semitic and Chinese writing systems—both of which have undergone extensive evolutionary stages—suggests that such patterns emerge from the interaction between the human sensory and higher cognitive systems. This hypothesis is supported by findings showing that grapho-phonemic systematicity is maximized when the salient features of an orthography are considered. For example, systematicity in Hangul reached a correlation of 0.6 when letters were analysed based on strokes, reflecting the system’s design principles [2]. Similarly, English achieved its highest grapho-phonemic systematicity when phonemes were analysed using criteria specified by Harm and Seidenberg [6,11].

In this paper, I investigate the behavioural basis of grapho-phonemic systematicity. By examining three types of systematicity—English as a representative of Semitic orthography, Hangul as a representative of artificial orthography, and Chinese—the study hypothesizes that naïve participants will favour the grapho-phonemic systematicity resembling their native language. This will demonstrate that familiarity influences preference for systematicity, thereby solidifying the concept of grapho-phonemic systematicity as a reflection of human behavioural patterns.

The study included participants with diverse linguistic backgrounds: English, Korean, and Chinese as their first languages. In the survey, participants were asked to select one of three given letter pairs that they believed corresponded to a given sound pair. Each of the three letter pairs embodied a distinct type of grapho-phonemic systematicity—English, Korean, or Chinese. The letter shapes were derived from various archaic writing systems. It is hypothesized that English speakers will predominantly choose the letter pair representing English-type grapho-phonemic systematicity, Korean speakers will favour the Korean-type pair, and Chinese speakers will select the Chinese-type pair.

## 2. Methodology

### 2.1. Designing experiment materials

For the purpose of the experiment, I first designed fictitious letter-sound correspondences. The primary focus of this research is on the distances between pairs of written symbols (and their associated sounds), rather than establishing a complete system with a one-to-one correspondence between individual letters and phonemes.

The phonemes selected for the experiment were those commonly found in the native languages of the target participants. Among all pairwise phonetic distances measured using various methods, 10 cases were selectively chosen to ensure a balanced distribution of distances. Using Euclidean distance [1], the selected phonetic distances were evenly distributed within a range of 1 to 2.24 (maximum value) (Table 1).

**Table 1:** The pairwise distances between two phonemes measured by four different methods.

No.	Phoneme 1	Phoneme 2	Distance measure			
			Euclidean	Cosine	Jaccard	Feature edit
**1**	g	s	2.24	1	1	5
**2**	h	m	2.24	1	1	5
**3**	k	s	2	1	1	4
**4**	p	h	2	1	1	2
**5**	b	k	1.73	0.59	0.75	3
**6**	p	g	1.73	0.59	0.75	3
**7**	b	m	1.41	0.33	0.5	2
**8**	h	s	1.41	0.5	0.67	2
**9**	k	g	1	0.18	0.33	1
**10**	p	b	1	0.18	0.33	1

**Note:** The distances measured using Euclidean distance were highly correlated with other phonetic metrics, including cosine distance (*r* = .97, *p* < .0001), Jaccard distance (*r* = .97, *p* < .0001), and feature edit distance (*r* = .90, *p* < .0001).

To eliminate any influence from participants’ prior knowledge of letter shapes or writing systems, the symbols were sourced from several archaic writing systems. Specifically, 17 Phoenician, 27 Aramaic, 54 Old Hungarian, and 46 Mkhedruli symbols were used for the study. To select the most suitable symbol pairs corresponding to the chosen sound pairs, 10 symbol pairs (20 symbols) were randomly selected and matched with the sound pairs. The pairwise distances between the symbols were then calculated, and the correlation between these distances and the phonemic distances was assessed. Using Python 3.8, an algorithm iteratively refined the selection by saving the phonemic and symbol lists along with the correlation coefficient, but only if the result outperformed the previous calculation. Ultimately, 10 symbol pairs that maximized the correlation with their corresponding phonemic pairs were chosen (Table in S1 Table).

Three distinct sets of materials were designed to highlight significant grapho-phonemic systematicity, each based on a different symbol distance metric (Table in S1 Table). In Material 1, the pairwise symbol distances were measured using pixel count, a metric that maximizes grapho-phonemic systematicity in many Semitic languages, such as English [1]. In Material 2, the symbols were selected to achieve the highest correlation when their distances were measured using perimetric complexity [10], as used in Chinese [8]. In Material 3, the symbol distances were calculated using Hausdorff distance [9], as used in Hangul, the Korean writing system [1]. In each material set, L1 and L2 correspond to P1 and P2, respectively, to achieve maximum systematicity.

Table 2 confirms that the English-type, Chinese-type, and Korean-type materials each exhibit robust grapho-phonemic systematicity when evaluated using their respective symbol distance metrics: pixel count, perimetric complexity, and Hausdorff distance. Each material demonstrates significant systematicity exclusively with the intended metric and fails to exhibit systematicity when alternative metrics are applied. This is a challenging goal to achieve because symbol distance metrics are not entirely independent in their mechanisms. For example, symbol pairs with high values for perimetric complexity often also show high values when measured by pixel count. Pixel count, in particular, can capture typologically distinct symbols with additional strokes, which are more specifically targeted by Hausdorff distance.

**Table 2:** Cross-validation of grapho-phonemic systematicity (Pearson’s *r* and Spearman’s *rho*).

	Systematicity	Material #1 “English-type”	Material #2 “Chinese-type”	Material #3 “Korean-type”
**Pixel Count**	*r* (p-value)	0.98 (<.0001)	0.45 (0.19)	0.62 (0.07)
	*rho* (p-value)	0.99 (<.0001)	0.34 (0.33)	0.86 (0.001)
**Perimetric Complexity**	*r* (p-value)	0.48 (0.19)	0.91 (<.0001)	0.52 (0.15)
	*rho* (p-value)	0.39 (0.26)	0.98 (<.0001)	0.66 (0.04)
**Hausdorff Distance**	*r* (p-value)	0.37 (0.32)	0.55 (0.10)	0.96 (<.0001)
	*rho* (p-value)	0.43 (0.21)	0.54 (0.11)	0.98 (<.0001)

### 2.2. Online surveys

The participants for the online survey were recruited between 20 May 2024 and 30 August 2024. The study was approved by the Research Ethics Committee at York St John University under Application No. ETH2324−0279. Participants were assigned random IDs for identification purposes when completing the survey. Online written consent forms were collected, and participants could only proceed with the survey after clicking ‘Agree’ on the consent form (https://osf.io/vqxn2/?view_only=9cadcf6c4e5448389eb517041cdcf45d).

**Fig 1:**
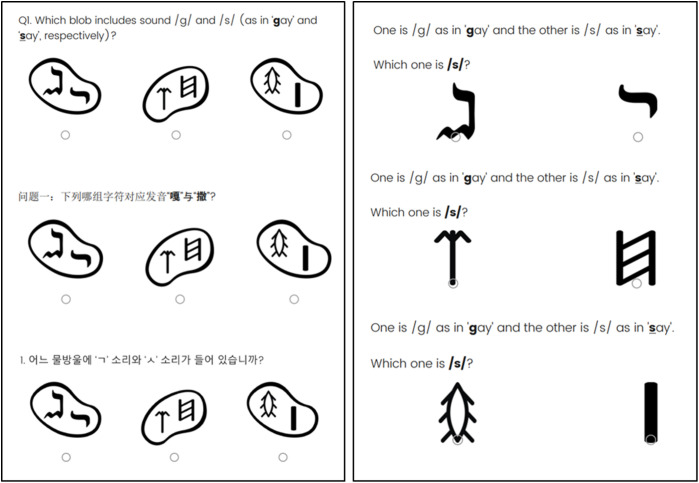
Example question translated in English, Chinese and Korean. Left: Three pairs of symbols were presented within blobs, each of which represents English-type, Chinese-type, and Korean-type grapho-phonemic systematicity. The naïve participants were asked to choose one of those blobs they considered appropriate for the given sound pairs. Right: The follow-up question if participants chose the first option (top), the second option (middle), or the third option (bottom). The participants were asked to link individually given symbols to the given sounds.

The online survey was distributed in three countries: UK, South Korea, and China. The survey questions were translated in the respective language. In the survey, the participants were asked to choose a set of symbol pair they think appropriate for the given sound pairs (Fig 1). As noted earlier, the research investigates participants’ sensitivity to the pairwise distance between two symbols, rather than requiring them to establish a one-to-one letter-sound correspondence.

For the identical phonetic pair, three symbol pairs from Table in S1 Table were presented—English-type, Chinese-type, and Korean-type. To minimise influence from letter arrangement, I put symbols in a blob so that participants do not automatically align symbols in spatial order.

The participants were firstly asked to choose the letter pair that may correspond to the given phoneme pair. Upon their choices, the survey led them to choose specifically which letter sounds which phoneme. If they click the first blob for the given phoneme pair/g/-/s/ (Fig 1, left), the next question specifically asked which symbol has/g/ and/s/, respectively (Fig 1, right). This sequential approach is expected to minimise the impact of any spatial ordering assumptions, especially from the word ‘respectively’ in the English version survey. Each participant was exposed to 20 questions in total.

For English version survey, example words were given along with the target phonemes considering English letter can have multiple different pronunciations [12]. For Korean version, there was no need to provide examples as a Korean letter exclusively indicates a phoneme. In Chinese, the symbols represented syllables rather than phonemes (see Fig 1).

Due to the conditional branching structure of survey questions and the need to maintain systematic correspondence between phoneme pairs and systematicity types, question order was not randomized across participants.

All distance calculations were performed using custom Python implementations, with complete code, materials, and data available at https://osf.io/vqxn2/?view_only=9cadcf6c4e5448389eb517041cdcf45d.

## 3. Results

**Fig 2:**
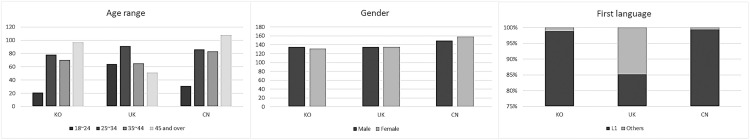
Visualised demographic distribution in each country.

A total of 845 participants were recruited online from South Korea (266), the UK (271), and China (308). Each participant received £6 for completing the survey within 3–10 minutes and passing an attention check. The demographic distribution of participants from each country is presented in Fig 2.

The vast majority of participants were native speakers of their respective target languages (UK: 84%, Korea: 99%, China: 99%). In the UK group, first languages included English (228), Hindi/Indian languages (20), other European languages (15), African languages (5), and Chinese (3). Korean participants primarily spoke Korean (263), with minimal representation of Japanese (1) and Thai (1). Chinese participants spoke Mandarin (302), Cantonese (2), or English (2), with two speaking other languages.

It was hypothesized that participants would show a preference for the grapho-phonemic systematicity associated with their first language—British participants would favour English-type systematicity, Chinese participants would favour Chinese-type systematicity, and South Korean participants would favour Korean-type systematicity. Responses were recorded based on whether participants selected the series of symbol pairs as intended in the survey design.

**Fig 3:**
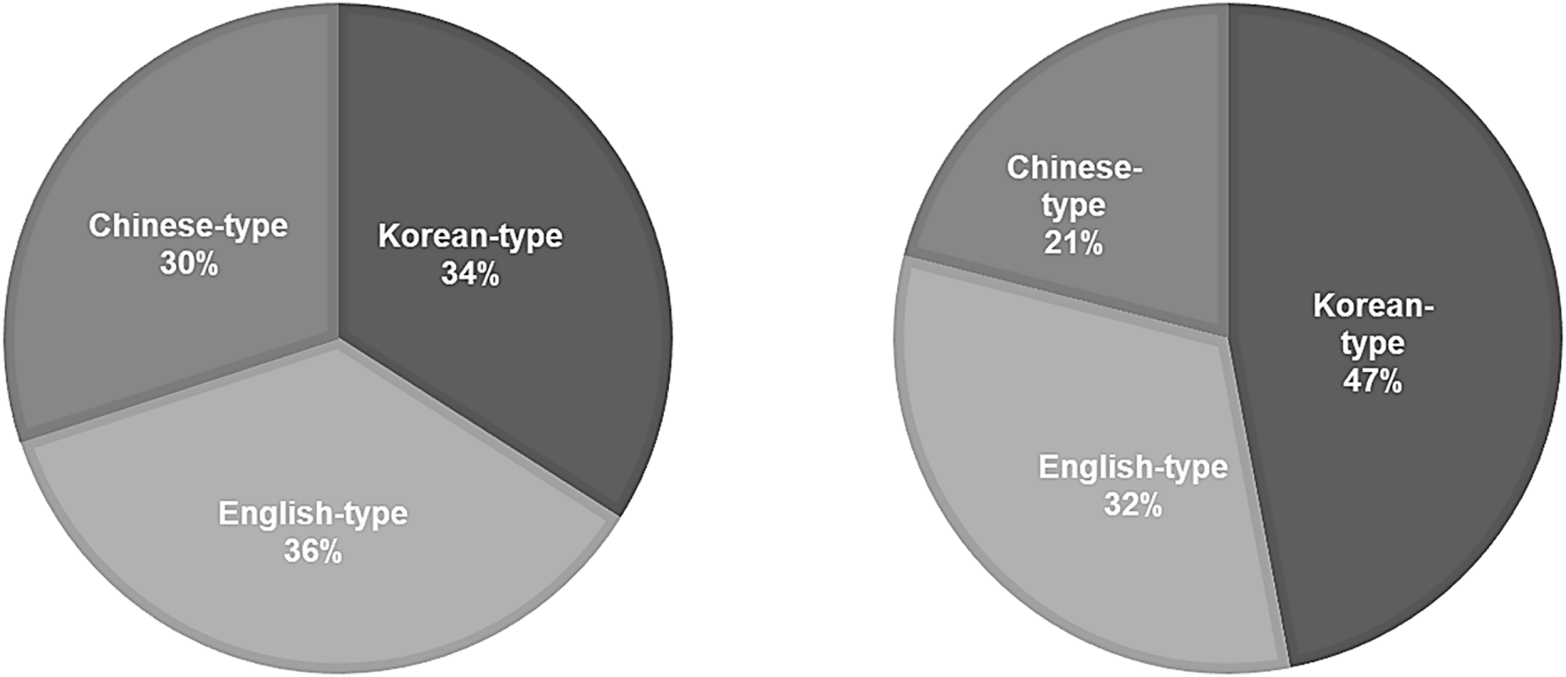
Probability of participants selecting the ‘intended’ grapho-phonemic systematicity designated for their group (left) and the probability of participants selecting any ‘preferred’ grapho-phonemic systematicity regardless of designation (right).

As shown in Fig 3, the average probability of participants selecting the designated type of grapho-phonemic systematicity across the series of tasks remained close to chance levels, at approximately 30% for each group (Fig 3, left). However, the probability of selecting Korean-type systematicity was higher across all participant groups, regardless of their first language (Fig 3, right).

**Fig 4:**
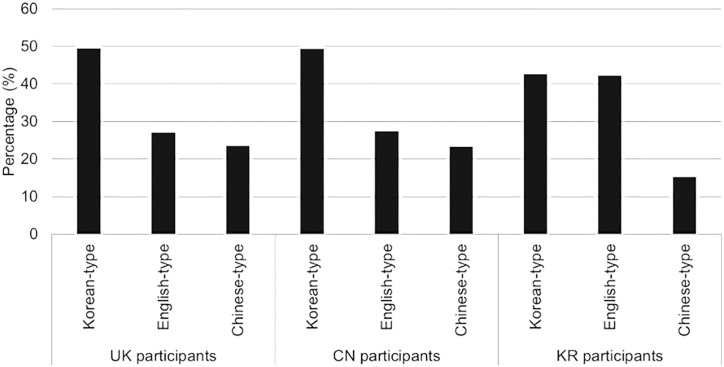
Proportion of ‘preferred’ grapho-phonemic systematicity by each group.

More detailed findings are illustrated in Fig 4. Notably, both British and Chinese participants demonstrated a strong preference for Korean-type grapho-phonemic systematicity, followed by a significant gap before favouring English-type systematicity. In contrast, South Korean participants exhibited an equal preference for Korean-type and English-type systematicity, indicating no particular preference for Korean-type systematicity. Across all three groups, Chinese-type systematicity was the least preferred.

**Fig 5:**
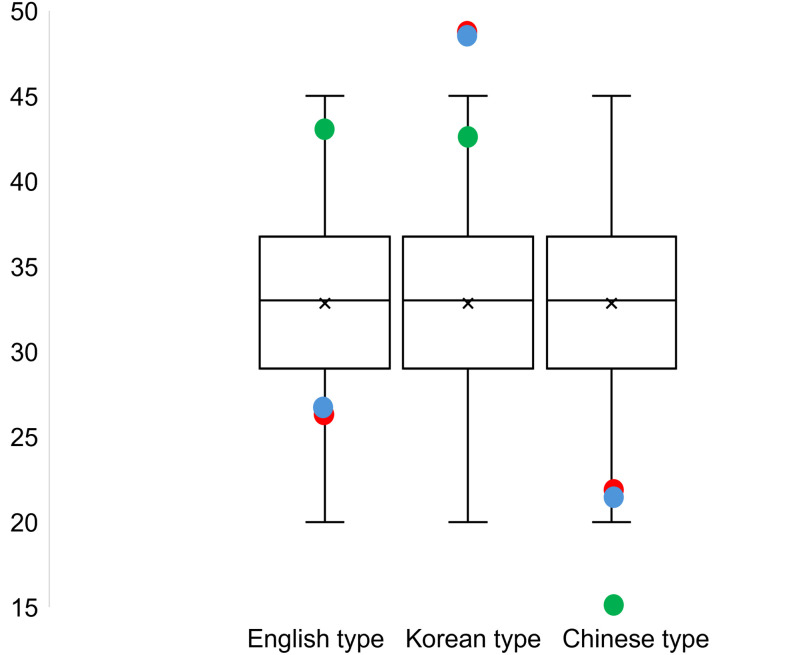
Monte Carlo permutation test results compared with the observed data. Red dot represents the preferences of British participants, blue dot represents the preferences of Chinese participants, and green dot represents the preferences of Korean participants.

A Monte Carlo permutation test was conducted to verify the statistical significance of the observations [13]. The computer simulation involved randomly selecting one of the three types of grapho-phonemic systematicity 10,000 times. The results are represented as box plots in Fig 5. Data points located outside the boxes indicate that the findings are statistically significant and not attributable to random chance.

The patterns observed in British (red) and Chinese (blue) participants, who displayed a preference for Korean-type systematicity, were noticeably beyond the chance-level probability (Fig 5). This highlights a stronger preference for Korean-type grapho-phonemic systematicity among non-Korean participants. Additionally, it is worth noting that Korean (green) participants did not prefer Chinese-type systematicity, which was the least favoured option across all three groups.

Table 3 illustrates correlations between participants’ selection patterns. Notably, preference for Korean-type systematicity consistently negatively correlates with English-type systematicity across all groups—KR preferring English and KR preferring Korean (*r* = −.67, *p* < .05); CN preferring English and CN preferring Korean (*r* = −.79, *p* < .01); UK preferring English and UK preferring Korean (*r* = −.89, *p* < .001). This suggests that competing forces may operate between preferences for Korean-type and English-type systematicity.

**Table 3:** Correlation matrix with p-values. * p < .05, ** p < .01, *** p < .001. KR, CN, and UK indicate participants from Korea, China, and the United Kingdom, respectively. Korean, Chinese, and English refer to systematicity types. For example: “KR preferring Korean” indicates Korean participants who preferred Korean-type systematicity; “KR selecting Korean (intended)” indicates Korean participants who selected Korean-type systematicity as intended by the experimental design.

	KR preferring Korean	KR preferring English	KR preferring Chinese	KR selecting Korean (intended)	CN preferring Korean	CN preferring English	CN preferring Chinese	CN selecting Chinese (intended)	UK preferring Korean	UK preferring English	UK preferring Chinese	UK selecting English (intended)
**KR preferring Korean**	1.00***											
**KR preferring English**	−0.67*	1.00***										
**KR preferring Chinese**	−0.72*	−0.03	1.00***									
**KR selecting Korean (intended)**	0.03	0.24	−0.27	1.00***								
**CN preferring Korean**	0.73*	−0.90***	−0.15	−0.07	1.00***							
**CN preferring English**	−0.49	0.77**	−0.05	0.25	−0.79**	1.00***						
**CN preferring Chinese**	−0.60	0.54	0.31	−0.20	−0.67*	0.09	1.00***					
**CN selecting Chinese (intended)**	−0.21	0.14	0.16	0.25	−0.32	0.20	0.27	1.00***				
**UK preferring Korean**	0.30	0.29	−0.66*	0.66*	0.03	0.22	−0.31	−0.22	1.00***			
**UK preferring English**	−0.37	−0.14	0.62	−0.54	−0.10	−0.02	0.18	0.09	−0.89***	1.00***		
**UK preferring Chinese**	0.24	−0.25	−0.09	−0.09	0.16	−0.38	0.20	0.24	0.02	−0.47	1.00***	
**UK selecting English (intended)**	0.30	−0.02	−0.38	−0.50	0.10	−0.36	0.28	−0.43	0.18	−0.33	0.36	1.00***

For each question, preference patterns were similar between Chinese and Korean participants. The two groups showed highly correlated preferences for Korean-type systematicity (*r* = .73, *p* < .01) and for English-type systematicity (*r* = .77, *p* < .01). Chinese and Korean participants show similar response patterns, suggesting a potential influence of Sino-sphere orthographic culture.

## 4. Discussion

### 4.1. Preference of certain type of grapho-phonemic systematicity

Based on the psychological principle that people tend to favour what they are familiar with [14], I hypothesized that each group of participants would prefer the sound-symbol mapping tendencies found in their own language—English-type for English speakers, Chinese-type for Chinese speakers, and Korean-type for Korean speakers. However, the results revealed that participants across all groups preferred the Korean-type grapho-phonemic systematicity, regardless of their language background. These unexpected findings suggest that participants’ choices involved a cognitively higher process that overrode a simple mere-exposure effect.

It is crucial to differentiate between systematicity as a product of natural selection, as suggested by findings from previous studies [1,5], and systematicity as a cognitive foundation for selection, as demonstrated in the current behavioural experiment. The former posits that collective behavioural patterns arise through repeated communication, involving numerous trials and errors over time. In contrast, the latter highlights outcomes where participants made choices from predetermined options. This paper’s discussion will centre primarily on the latter perspective.

The findings from the current study suggest that the participants demonstrated general ‘proportional reasoning’ [15], where individuals use a relational approach to represent quantities without relying on precise calculations. Tasks might include for example, estimating the height of water in a cylinder with a 5 cm diameter after transferring it from a cylinder with a 10 cm diameter, or approximating the time of arrival by bicycle for a distance typically covered by car. These tasks do not necessarily require numerical evaluation. For instance, how would one decide the appropriate punishment for two criminals: one who stole a goat from a neighbour and another who committed murder?

This type of formal inference serves as a mental model for logical thinking [16] and depends on the ability to use analogical reasoning, mapping one’s understanding onto relations learned in other contexts [17]. Spatial Layout Model [15] suggests that proportional reasoning processes may involve the parietal and dorsal areas of the brain, which handle spatial “where” information rather than visual “what” information. These processes engage elements that cannot be directly visualized yet require experiential imagery [18]. Judging the last example above particularly relies on ‘intuitive’ proportional reasoning, as numerical scales are unavailable [19]—the severity of punishment proportionally corresponds to the severity of the crime.

Participants in this study clearly preferred proportional mapping when associating visual symbols with sounds. They consistently favoured Korean-type systematicity, where similar sounds are linked to topologically similar symbols, as measured using the Hausdorff distance [9]. Although all three types of experiment materials were designed to align with intuitive proportional decision-making, I propose that the preference for Korean-type systematicity may stem from the presence of clear evidence supporting selection.

The symbols for this systematicity (Material 3, Table in S1 Table) provide logical explanations for participants’ choices—such as one part of a symbol being more elaborated or having more lines than the other (e.g., ¬/g/ compared with ㅋ/k/, and ㅏ/a/ compared with ㅑ/ja/), which proportionately corresponds to the phonetic distance they were given. I argue that this tendency to “pursue a reason for selection” or “seek logic based on evidence” likely influenced participants’ decisions, with modern education serving as the foundation for this reasoning process. Since the Industrial Revolution, certain forms of modern logic—such as reductive scientific frameworks, numerical and mathematical reasoning, and legal interpretation, etc.—have been systematically integrated into institutional education and public common sense and steered the way we reason proportion. This may let the participants prefer mapping based on Korean-type systematicity where proportional distance is represented through noticeable visual difference.

An alternative interpretation of the findings relates to the interdependence of visual distance metrics. Although Korean-type materials (Material 3 in Table in S1 Table) were designed to maximize systematicity using Hausdorff distance, they also demonstrated relatively strong systematicity when measured by pixel count (Table 2). As noted in our methodology, these distance metrics are not entirely independent—symbols with very short Hausdorff distances (indicating topological similarity) may also use similar amounts of ink, creating convergent effects across metrics.

This interdependence suggests that the universal preference for Korean-type systematicity may not solely reflect the proportional reasoning processes discussed above. Instead, participants may have been attracted to symbol pairs that demonstrate robust systematicity across multiple visual dimensions simultaneously. From this perspective, their choices could be driven by cognitive confidence arising from convergent evidence of systematicity. This interpretation would suggest that humans intuitively seek symbol-sound mappings supported by multiple forms of visual evidence, potentially explaining why Korean-type materials were preferred even by non-Korean participants.

### 4.2. Observations from the Korean participants

Interestingly, the Korean native group exhibited the least preference for Korean-type grapho-phonemic systematicity compared to the other groups, demonstrating a comparable level of preference for English-type systematicity. Since no interviews were conducted with the Korean participants, the underlying reasons for this phenomenon remain unclear. However, as a native Korean, I hypothesize that this tendency may stem from their awareness of the historical background and implications of Hangul’s creation. Recognizing that Hangul is the only writing system intentionally designed to maximize systematic correspondence between sounds and symbols might have influenced Korean participants to project this awareness onto the experiment. They may have consciously avoided selecting the systematicity that resembled their native writing system, opting instead for the one observed in English. In contrast, participants from the other two groups, who likely lacked such specific expectations regarding writing systems, appeared to select what they perceived as the ideal connection between letter shapes and sounds.

Additionally, Korean participants chose English-type systematicity as an alternative to the Korean type, suggesting that English-type systematicity ranks second in preference after the Korean type. All three groups showed the least preference for Chinese-type systematicity. This indicates that participants either least preferred or were least sensitive to pairing phonemes with symbols based on visual complexity (perimetric complexity). The universal dis-preference for Chinese-type systematicity suggests that this metric may not align with intuitive symbol-sound mapping preferences, despite evidence of this systematicity in Chinese orthography systems [7,8].

### 4.3. Further research

I investigated if the three distinct grapho-phonemic systematicity found in the previous series of research is also observed through behavioural experiment. Unlike expectation, Korean-type systematicity was most favoured by the 845 participants regardless of their first language. The findings indicate that there may be certain preference in symbol-sound mapping. A possible explanation was provided—the modern institute have steered human proportional reasoning to a certain type of logic and Korean-type systematicity represents it more clearly than the other types of grapho-phonemic systematicity.

To investigate this assumption, it is necessary to replicate the experiment with pre-school or early-school children, as they have had limited exposure to institutional education and are less influenced by formalized proportional reasoning. These younger participants represent a population whose cognitive processes are less shaped by modern educational frameworks, allowing researchers to observe whether the preference for grapho-phonemic systematicity arises naturally or is primarily a by-product of acquired reasoning patterns.

By examining their responses, we can determine whether the observed preference for Korean-type systematicity reflects an inherent cognitive bias or is a result of societal conditioning. Additionally, such a study would provide insights into the developmental stages of reasoning and how exposure to institutional education gradually shapes cognitive preferences for symbolic systems. Comparing these findings with adult participants could further clarify the role of education and environmental influences in shaping perceptions of systematicity.

The findings show a disconnect between how writing systems historically evolved and what modern humans prefer. Why were three different types of grapho-phonemic systematicity observed in Semitic, Chinese, and Korean writing systems if they have not evolved to increase the systematicity in the respective way? It should be pointed out that those fictitious writing systems designed for entertaining purposes did not return any systematicity, implying that the grapho-phonemic systematicity is the results of actual human behaviour.

This line of research must require diachronic investigation through the history of each writing system. A previous study explored if ancient writing systems—Phoenician, Nabataean, Early Arabic and Aramaic—returns any systematicity but failed [6]. This was attributed to authenticity of the recovered letter shapes and phonemes, which may be not perfectly reliable [20–21], and to the lack of fonts that limited the scope of the analysis.

The inability to reveal grapho-phonemic systematicity in several ancient orthographies implies three possibilities. First, grapho-phonemic systematicity exists in the ancient writing systems examined, but the chosen letter shapes and sounds may not have been accurately represented. Second, it is possible that systematicity did not exist in the early stages and only developed later, which could explain why modern orthographic systems exhibit such systematicity. From this perspective, one could assume that human orthographic systems have evolved to develop systematic relationships between letter shapes and sounds. Finally, ancient writing systems may exhibit a different form of systematicity that requires a distinct approach, warranting further methodological exploration.

Future research could benefit from incorporating eye-tracking to complement the survey-based approach by providing real-time data on participants’ attention patterns to specific visual features. Such data could also reveal temporal dynamics of decision-making, showing whether preferences emerge from immediate visual processing or require more deliberative comparison of symbol pairs. Additionally, multivariate analyses such as mixed-effects modelling could disentangle whether systematicity preferences reflect perceptual, cultural, or individual difference factors.

Last but not least, what practical benefits of grapho-phonemic systematicity are there in terms of learning and acquisition? One behavioural research showed that the Korean veridical letter-sound association did not noticeably facilitate learning the writing system, compared to random letter-sound association condition [6,22]. The participants were able to acquire the given associations in both the veridical and random conditions. Further exploration will only be possible once the mechanism of grapho-phonemic systematicity is firmly established.

## 5. Conclusion

The findings from this research suggests that there may be universal cognitive principles underlying how we map visual symbols to sounds, challenging the traditional view that writing systems develop arbitrarily or are purely culturally determined. The results were partially explained by proportional reasoning applied to mapping symbol-sound connection, which might have steered to prefer a certain type of connection through formal education. Less preference for Korean-type systematicity among the Korean participants suggests that meta-knowledge about writing systems might affect how people perceive and judge the systematicity between symbols and sounds. These findings open the door to more questions than answers, highlighting the vast potential for further exploration in this area. I hope future studies will build on this work and contribute to the continued expansion of this field of study.

## Supporting information

S1 Table Three material sets designed to maximize different types of grapho-phonemic systematicity. (DOCX)
