# Supplementary material for: Universal preference for Korean-type grapho-phonemic systematicity: a cross-cultural study of sound-symbol mapping in English, Chinese, and Korean speakers
Source: PLoS One. 2025 Aug 29;20(8):e0330674. doi: 10.1371/journal.pone.0330674 (PMC12396722; doi:10.1371/journal.pone.0330674)
Supplement: S1 Table — (DOCX) [file pone.0330674.s001.docx]

**S1 Table.** Three material sets designed to maximize different types of grapho-phonemic systematicity.

|  | **Phonetic distance** | | | **Material #1 “English-type”** | | | | **Material #2 “Chinese-type”** | | | | **Material #3 “Korean-type”** | | | |
| --- | --- | --- | --- | --- | --- | --- | --- | --- | --- | --- | --- | --- | --- | --- | --- |
| **No.** | P1 | P2 | Euclidean distance | Origin | L1 | L2 | Pixel  count | Origin | L1 | L2 | Perimetric complexity | Origin | L1 | L2 | Hausdorff distance |
| **1** | g | s | 2.24 | Old Hungarian | 𐲌 | 𐲬 | 18510 | Old Hungarian | 𐲡 | 𐳺 | 9.34 | Old Hungarian | 𐲯 | 𐲁 | 75.29 |
| **2** | h | m | 2.24 | Old Hungarian | 𐲧 | 𐲯 | 19392 | Aramaic | ר | ש | 7.09 | Aramaic | א | ר | 83.00 |
| **3** | k | s | 2.00 | Old Hungarian | 𐲘 | 𐲱 | 13197 | Mkhedruli | Ⴢ | ჶ | 4.76 | Old Hungarian | 𐲙 | 𐲃 | 54.59 |
| **4** | p | h | 2.00 | Old Hungarian | 𐲠 | 𐲃 | 12138 | Aramaic | א | ן | 4.05 | Old Hungarian | 𐲭 | 𐲌 | 50.04 |
| **5** | b | k | 1.73 | Old Hungarian | 𐲡 | 𐲝 | 8541 | Aramaic | ז | ב | 2.86 | Aramaic | ה | ל | 36.07 |
| **6** | p | g | 1.73 | Phoenician | 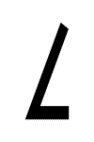  U+1090B | 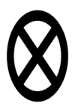  U+10908 | 8802 | Mkhedruli | ჿ | ჳ | 1.98 | Mkhedruli | ჳ | ლ | 35.51 |
| **7** | b | m | 1.41 | Aramaic | ם | ץ | 6240 | Phoenician | 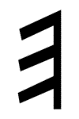  U+10904 | 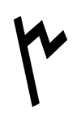  U+10911 | 0.69 | Aramaic | כ | פ | 32.02 |
| **8** | h | s | 1.41 | Old Hungarian | 𐲪 | 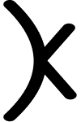 | 4512 | Phoenician | 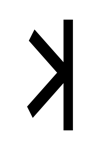  U+1090A | 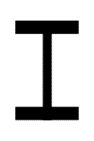  U+10906 | 0.21 | Old Hungarian | 𐲂 | 𐲨 | 27.73 |
| **9** | k | g | 1.00 | Mkhedruli | გ | ყ | 3 | Mkhedruli | ბ | ჰ | 0.01 | Mkhedruli | ჲ | ი | 8.94 |
| **10** | p | b | 1.00 | Mkhedruli | ჱ | ქ | 3 | Old Hungarian | 𐲚 | 𐳤 | 0.01 | Mkhedruli | ი | ო | 10.00 |
